# A crime case involving a mixture of three individuals solved using a sample from the daughter of the person of interest

**DOI:** 10.1007/s00414-025-03472-8

**Published:** 2025-03-27

**Authors:** Nelly Alexandra Gómez, Ana Patricia Robles Nieto, Ginna Chacón, Rocio Lizarazo, Thore Egeland

**Affiliations:** 1 Grupo de Genética Forense, Instituto Nacional de Medicina Legal y Ciencias Forenses, Calle 7A N°12 A 51, Bogotá, Colombia; 2 Faculty of Chemistry, Biotechnology and Food Science, Norwegian University of Life Sciences, Aas, 1433 Norway; 3 Forensic Genetics Research Group, Department of Forensic Sciences, Oslo University Hospital, Oslo, Norway

**Keywords:** Evaluation of mixtures with related contributors, Complex mixture profiles, relMix and EuroForMix software, Impact of using peak heights, Colombian forensic cases

## Abstract

A forensic genetics laboratory may well receive requests that are out of the ordinary and pose challenges like the one presented in this case. A mixture from three individuals was analysed from a crime scene. Reference samples were obtained from two individuals. The Person Of Interest (POI) was not available, only a putative daughter. The analysis of Y and X-STR markers indicated that the contributors to the bloodstain mixture were two males and one female. The hypotheses for the calculation of the Likelihood Ratio (LR) were specified and evaluated using autosomal STR markers. The calculations were done using the open and freely available programs relMix and EuroFormix. The software relMix can deal with arbitrary family relationships between contributors while EuroFormix only accommodates pairwise relationships. In this case both programs were applicable. EuroFormix models peak heights resulting in higher LR, as expected. Ultimately, results from both programs identified the POI, leading to the resolution of the case.

## Introduction

According to figures from the National Institute of Legal Medicine and Forensic Sciences, Colombia, during the years 2022 and 2023 approximately 17 bodies were found in the city of Bogotá, in the fetal position and in garbage bags. These crimes are attributed to micro-trafficking criminal gangs in Bogotá. The Genetics Laboratory of the National Institute of Legal Medicine has headquarter in Bogotá and deals with such cases and enters genetic profiles of unidentified persons into the Genetic Profile Bank of Missing Persons. We report on one of these cases and suggest how similar crimes can be analysed.

In mid-2023, our laboratory received a challenging case: The prosecutor’s office discovered three male bodies wrapped in garbage bags inside a recycling vehicle. During a raid on a nearby house suspected of being used for dismembering bodies, 11 bloodstained pieces of evidence were recovered, along with a roll of black plastic. The plastic matched the characteristics of the material used to wrap the three bodies, with bloodstains visible on its ends.

Among these pieces of evidence, unique bloodstains containing the profiles of the male bodies were identified, while others showed mixtures of their profiles. Only one bloodstain on the plastic revealed a mixture containing the profiles of two male bodies and an unknown female. The prosecutor of the case asked if the female contributor was the woman reported as missing days earlier. She was last seen near the place where the bodies were found, and her presumed daughter was available for genotyping. The purpose of this article is to illustrate the analyses and the probabilistic approach. It is well known that it can be difficult to determine the number of contributors to a mixture based on autosomal markers. However, X and Y markers can help and also indicate presence of males and females [1] as exemplified in this report. Moreover, cases involving related contributors to a mixture are potentially challenging.

The open and freely available software relMix and EuroForMix were used to solve the case, and we also comment on how similar cases can be approached.

## Methods

The objective of this study is to determine whether the genetic profile of a woman reported missing matches the cellular mixture recovered from the bloodstain. The last known sighting of the missing woman was when she reportedly entered a house near the location where the bodies were found. This analysis is constrained to the blood sample from her daughter. Thus, it is crucial to calculate, from a mixture of genetic profiles involving at least three individuals, the likelihood that the genetic profile of the missing woman, who is the biological mother of the daughter, is present in this mixture.

### Ethics

The study protocol and the use of genetic samples were conducted in accordance with standards of the National Institute of Legal Medicine and Forensic Sciences. Informed consent for the utilization of biological material from the daughter in this forensic study was obtained from the father, who legally represents the minor. The National Institute of Legal Medicine and Forensic Sciences ensures compliance with all relevant legal and ethical guidelines regarding the handling and analysis of genetic material in forensic investigations. Efforts were made to uphold the privacy and confidentiality of all individuals involved in accordance with institutional policies.

### DNA sampling, extraction and STR genotyping

The black plastic wrap sample underwent the extraction process using the Automate Express™ Forensic DNA Extraction System (Applied Biosystems™) with PrepFiler™ Forensic DNA Extraction Kits. All samples, including the reference samples from individuals labelled 1 and 2, and the daughter of the missing woman, were amplified with PowerPlex^®^ 21 System and PowerPlex^®^ Y23 System kits with a final reaction volume of 12.5 µL in a BioRAD C-100 thermocycler. Additional analyses of the X chromosome were performed using the Investigator Argus X-12 QS kit. For the reference sample of the missing woman’s daughter, typing was carried out with the PowerPlex^®^ Fusion System. The Applied Biosystems 3500xL genetic analyzer was used, and the results were evaluated with GeneMapper^®^ software version 3.1. The interpretation of the results complied with the criteria specified in the laboratory, including the reproducibility of the electropherograms, analytical thresholds, contributor proportions, and stochastic thresholds for each of the analyzed samples.

## Results

**Fig. 1:**
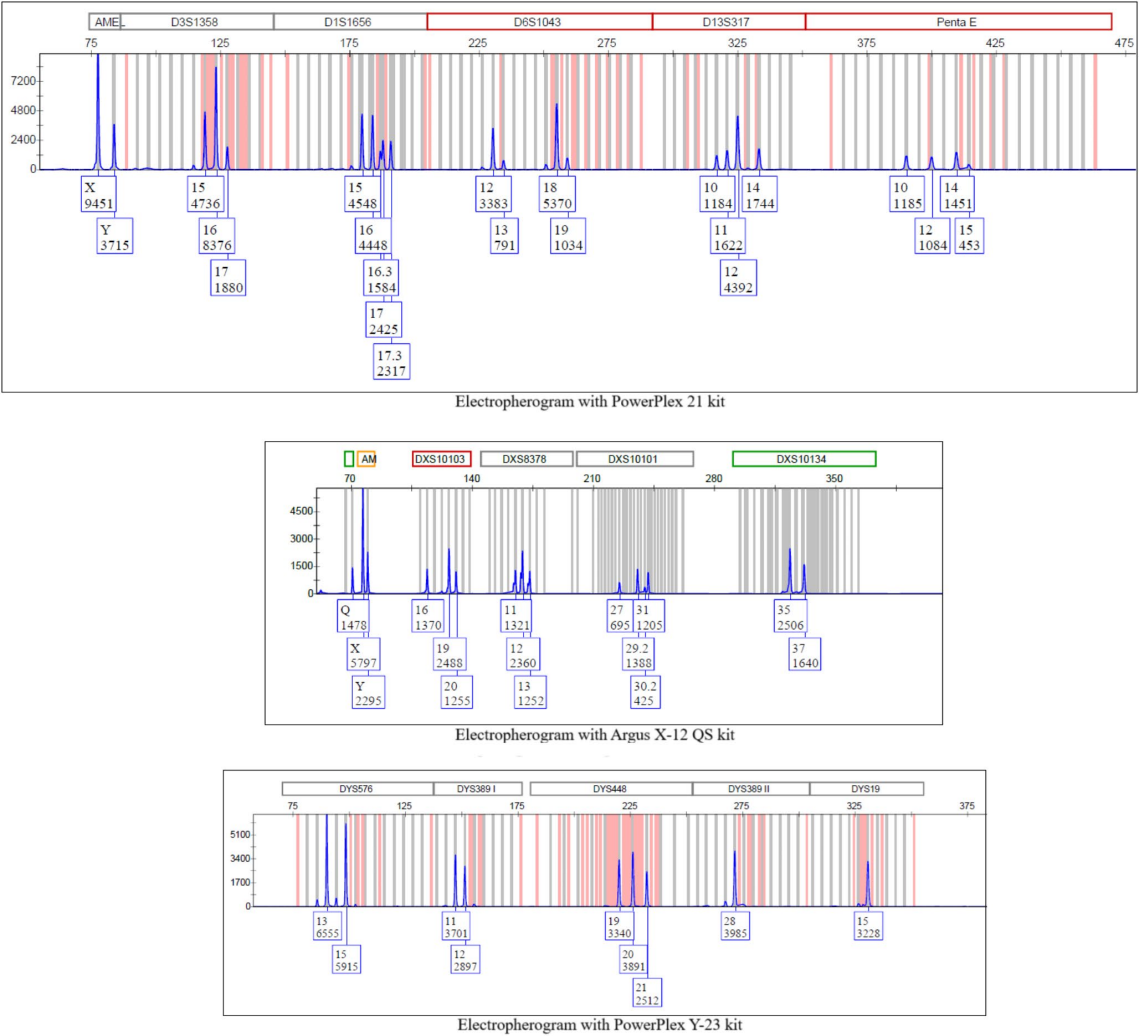
Electropherograms from the plastic wrap with PowerPlex 21, Argus X-12 QS and PowerPlex Y-23 kits

In the bloodstain, a DNA mixture from at least three individuals was detected: The genetic profiles of two male victims and at least one unknown individual, most likely female. The approximate female to male ratio was 2.5, which agrees well with the DNA quantification results (X/Y) = 2.64 (Fig. 1). By combining these results with Y chromosome markers, haplotypes from at least two unrelated male individuals were determined (data not shown).

**Fig. 2:**
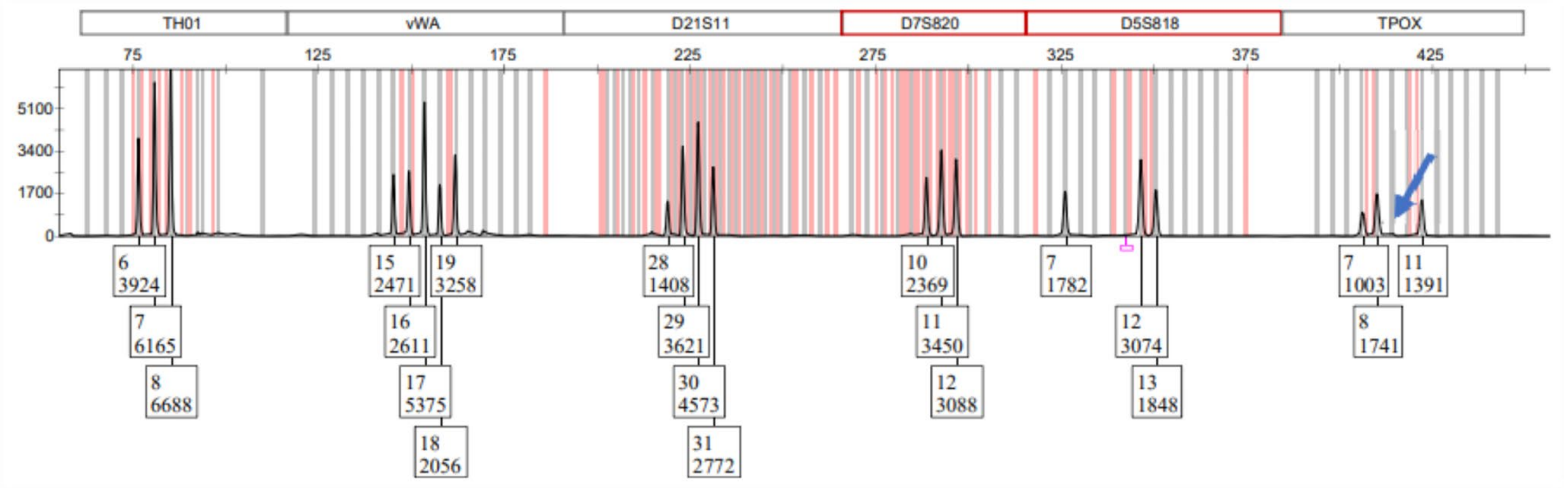
Electropherogram showing allelic loss of allele 9 for the TPOX genetic system in male individual 2

The results obtained from the X-chromosomal markers confirmed the Y-chromosome analysis, indicating that the mixture is composed of cells from at least three individuals, two male and one female, the latter being the major contributor [2]. One of the individuals presented a duplication (19, 20), explaining why three alleles (19, 20, 21) are observed and confirming the contribution of two male individuals. Additionally, it is observed that the predominant female profile shares an allele in all the biparental genetic systems and in the clusters of the X-STRs analyzed with the daughter of the missing woman. Furthermore, a drop-out of allele 9 was observed for TPOX for one of the male contributors, as shown in Fig. 2. This drop-out is not unexpected based on internal validation, the contribution from the other donors, and the findings from the other evidence analyzed in the case, where the clean and complete profile of this individual was detected.

### Statistical calculations

Based on the above analyses, we assume that there are three contributors to the mixture, one female and two males. This is compatible with the male components in the mixture found in the garbage bags.

**Fig. 3:**
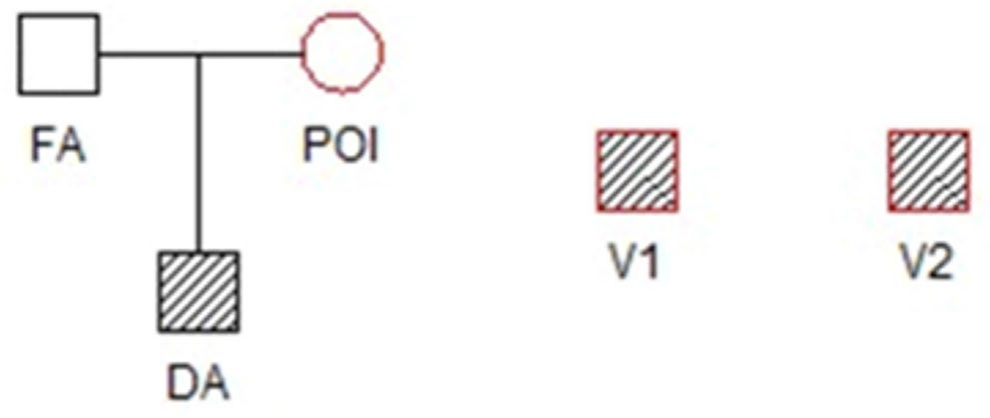
Illustration of the prosecutor hypothesis

The prosecutor hypothesis is:

$$H_P: \mathrm{POI}, \mathrm{~V} 1 \text { and } \mathrm{V} 2 \text { contributed } \text { to } \text { the } \text { mixture } \mathrm{R}$$

where POI (The Person Of Interest (POI), a female, ) is the mother of DA, see Fig. 3. The contributors to the mixture are indicated with red while genotyped individuals are hatched.

The defense hypothesis is:

$$H_D: \mathrm{~V} 1, \mathrm{~V} 2\ \text { and } \text { an } \text { unknown } \mathrm{NN} \text { contributed to the mixture } \mathrm{R}$$

All individuals are unrelated under *H*_*D*_. The *LR* can be written.

1 $$\:\frac{P\left(data\mid\:{H}_{P},R\right)}{P\left(data\mid\:{H}_{D},R\right)}=\frac{\sum\:_{{g}_{\text{POI}}}P\left(\text{POI}\mid\:\text{DA},V1,V2,{H}_{P},R\right)}{\sum\:_{{g}_{\text{NN}}}P\left(\text{NN}\mid\:V1,V2,{H}_{D},R\right)}$$

The sum in the numerator is over all genotypes consistent with the mixture and POI being the mother of DA. The sum in the denominator is the sum over all genotypes for NN consistent with the mixture. For the probabilistic calculations, the frequencies from the Andean region of the Colombian population were used for 13 CODIS markers [3], D2S1338 and D19S433 [4], D12S391 [5], PENTA E and PENTA D [6], D1S1656 [7] and D6S1043 [8].

### Example 1: LR calculation for the marker D8S1179

In this example we make all the simplifying assumptions, i.e., Hardy-Weinberg Equilibrium (HWE), no mutations, no drop-out or drop-in and no silent alleles.

The mixture is R = {13, 14, 15}. V1 is 13/14 and V2 is 14/14. Hence, under H_D_, NN must be 15/15, 14/15 or 13/15. Therefore, the denominator in the LR (1) is p_15_(p_15_ + 2p_14_ + 2p_13_), where p_13_, p_14_, and p_15_ denote the allele frequencies. Turning to the prosecutor hypothesis, POI must also be 15/15, 14/15 or 13/15. Since the genotype of DA is 15/15, the numerator is p_15_ + p_14_ + p_13_. Consequently,

$$\:LR=\frac{{p}_{15}+{p}_{14}+{p}_{13}}{{p}_{15}\left({p}_{15}+2{p}_{14}+2{p}_{13}\right)}.$$

Inserting the allele frequencies *p*_*13*_ *=* 0.333, *p*_*14*_ = 0.251 and p_15_ = 0.110, we get *LR =* 4.937. The above example, and similar calculations not included, merely serve as an illustration and a check of calculations using the software as described next.

### *LR* calculations using the software relMix

We use the software relMix v.1.3.5 [9, 10] for our LR calculations in this section. The main reason for using software is that derivations, like the one shown in the above example, may become difficult, and in practice impossible, if the simplifying assumptions made above are relaxed. In particular, we need to address drop-out. For the marker TPOX the individual V1, a contributor under both hypotheses, has the allele 9 not seen in the mixture. We specified a drop-out probability of 0.05 for V1 giving a total LR at 190,173, see Table 1. Obviously, it is difficult to assign the drop-out probability. Assumptions and sensitivity analyses are discussed later. Note that LR for the marker D8S1179 is 4.926, slightly reduced compared to 4.937 calculated above since the latter did not accommodate drop-out.

**Table 1:** LRs for RelMix and EuroForMix

Marker	LR relMix	LR EuroForMix
CSF1PO	1.344	1.463
D12S391	0.952	0.963
D13S317	4.849	5.118
D16S539	2.266	1.840
D18S51	1.158	2.720
D19S433	0.789	1.554
D1S1656	2.201	3.108
D21S11	1.208	1.324
D2S1338	1.780	2.487
D3S1358	1.304	1.536
D5S818	8.931	8.658
D7S820	0.843	1.278
D8S1179	4.926	4.801
FGA	0.495	1.417
PentaD	1.591	1.133
PentaE	4.360	4.052
TH01	1.437	1.337
TPOX	2.293	2.037
VWA	5.717	6.105
**Total**	190 173	5 060 746

### *LR* calculations using the software EuroForMix

The database of allele frequencies and reference profiles are the same as for the above results based on relMix. We used the following default settings of EuroForMix v4.0.8 [11, 12]: Analytical threshold (50), drop-out (0.05) drop-in (0.01) and stutter (beta priors). Table 1 gives the total LR as 5,060,746, roughly 27 times larger than relMix gave. This demonstrates the impact of utilizing peak height data.

## Results and discussion

Sensitivity analyses incorporating deviation from HWE, mutation, silent alleles, drop-out and drop-in were performed for relMix. As expected, these modifications reduce the LR, but the LR value stayed above 100 000 for realistic values. For instance, a drop out probability of 0.001 gives LR 1.824 105.

Model validation performed within EuroForMix did not indicate violations of the assumptions. Also, for EuroForMix, there are several parameters that can be varied. But sensitivity analyses did not reveal substantial changes. The total LR always stays above any reasonable threshold. We have a replicate of the mixture with peak heights for 17 of the 19 markers. Using also this replicate would roughly double the LR. We chose not to include this data in our main analysis to facilitate comparison with relMix.

Table 1 permits comparison between the programs and indicates the impact of using peak height data. The differences in LR values are not surprising for most markers. There are three markers (D19S433, D7S820, FGA) giving LR on either side of 1. These markers and D18S51 explain a substantial part of the difference in LR between the two programs. Obviously, peak height data should be used when possible. However, we are not aware of freely available software that can model peak height data while accommodating general family relationships between contributors to a mixture. In such cases, relMix can be used, but unfortunately peak heights cannot be accounted for in the current implementation.

Fortunately, methods are available to extend relMix to accommodate peak heights or for EuroFormix to allow for general pedigrees. EFMrep, is an extension of EuroForMix which enables the combination of STR DNA mixture samples from different multiplexes. In addition to calculating combined likelihood ratios and carrying out deconvolution, the software also includes the capability to specify several pairwise relationships between individuals [13].

Obviously, genotyping additional relatives of the victim, like the father (denoted FA in Fig. 3), would have given even stronger evidence. However, this was not deemed necessary, at least not initially, and would have delayed the case.

We included individuals V1 and V2 in both hypotheses. Other hypotheses, omitting one or both of V1 and V2, could have been considered, for example if the objective had been to question the contribution from suspects. However, our formulation was consistent with our mandate, to identify the third contributor, the mother of the daughter.

## Conclusion

In forensic genetics, encountering atypical cases necessitates a meticulous approach to probabilistic calculations. It is crucial to rigorously formulate the hypotheses formulated, ensuring that they accurately address the investigative queries. Selecting appropriate software tools for these calculations is equally paramount.

In this study, we faced the unique challenge of determining if one contributor to a three-person mixture corresponds to the biological mother reported missing by her daughter. Such scenarios are uncommon, warranting a detailed exploration of various probabilistic calculation methodologies. Notably, our analysis includes a comprehensive examination of allelic loss within the TPOX genetic system.

This case underscores the complexity and importance of advanced forensic genetic techniques in resolving challenging familial relationships and missing persons investigations. By illustrating our approach and findings in detail, we contribute to the broader forensic genetics community’s understanding and methodology refinement.

Our detailed probabilistic analysis enabled us to resolve this case effectively, highlighting the crucial role of forensic genetics in complex investigations. This study underscores the importance of advanced forensic genetic methodologies in elucidating intricate familial relationships and aiding in missing persons cases. By refining the methodology and enhancing understanding, we contribute valuable insights to the forensic genetic community, demonstrating new possibilities for analyzing complex cases.

## References

[1] Gomes I, Pinto N, Antão-Sousa S, Gomes V, Gusmão L, Amorim A (2020) Twenty Years Later: A Comprehensive Review of the X Chromosome Use in Forensic Genetics [Internet]. Vol. 11, Frontiers in Genetics. Frontiers Media S.A. 10.3389/fgene.2020.00926 10.3389/fgene.2020.00926 PMC7527635 33093840

[2] Bille T, Weitz S, Buckleton JS, Bright JA (2019) Interpreting a major component from a mixed DNA profile with an unknown number of minor contributors. Forensic Sci Int: Genet 40:150–159 30844683 10.1016/j.fsigen.2019.02.017

[3] Paredes M, Galindo A, Bernal M, Avila S, Andrade D, Vergara C, Rincón M, Romero RE, Navarrete M, Cárdenas M, Ortega J, Suárez D, Cifuentes A, Salas a, Carracedo A (2003) Analysis of the CODIS autosomal STR loci in four main Colombian regions. Forensic Sci Int 137(1):67–73 14550617 10.1016/s0379-0738(03)00271-8

[4] Porras L, Gen L, Beltrán T, Ortiz P, Sanchez-Diz A, Carracedo J, Henao (2008) Genetic polymorphism of 15 STR loci in central Western Colombia. Forensic Sci International: Genet Volume 2, Issue 1, e7 - e8 10.1016/j.fsigen.2007.08.004 19083781

[5] Gómez N, Jiménez M, Galindo A, Lizarazo R, Calderón G, Camargo M (1999) Análisis de Los loci D21S11 Y D12S391 En Población mestiza de Santafé de Bogotá, Colombia. IV Jornadas genética forense. Grupo español portugués de la ISFG. Gomera (España)

[6] Yunis J, García O, Cuervo A, Guio E, Pineda C, Yunis E (2005) Population data for powerplex 16 in thirteen departments and the capital City of Colombia. J Forensic Sci May 50, 3. 15932109

[7] Hill CR, Duewer DL, Kline MC, Sprecher CJ, McLaren RS, Rabbach DR, Krenke BE, Ensenberger MG,.Fulmer PM, Storts DR J.M. Butler Concordance and population studies along with stutter and peak height ratio analysis for the PowerPlex^®^ ESX 17 and ESI 17 Systems. Forensic Science International: Genetics, Volume 5, Issue 4, 269–275D.R. Storts 10.1016/j.fsigen.2010.03.014 20457109

[8] Hill CR, Duewer DL, Kline MC, Coble MD, Butler JM (2013) U.S. Population data for 29 autosomal STR loci, forensic sci. Int Genet 7:e82–e83 10.1016/j.fsigen.2012.12.004 23317915

[9] Dørum G, Kaur N, Gysi M (2017) Pedigree-based relationship inference from complex DNA mixtures. Int J Legal Med 131:629–641 28101646 10.1007/s00414-016-1526-x

[10] Elias Hernandis G, Dørum T Egeland (2019) RelMix: an open source software for DNA mixtures with related contributors. Forensic Sci Int: Genet Supplement Ser 7:221–223

[11] Benschop CCG, Nijveld A, Duijs FE, Sijen T (2019) An assessment of the performance of the probabilistic genotyping software EuroForMix: trends in likelihood ratios and analysis of type I & II errors, forensic sci. Int: Genet 42:31–38 10.1016/j.fsigen.2019.06.005 31212207

[12] Bleka Ø, Storvik G, Gill P (2016) EuroForMix: an open-source software based on a continuous model to evaluate STR DNA profiles from a mixture of contributors with artefacts. Forensic Sci Int Genet 21:35–44 26720812 10.1016/j.fsigen.2015.11.008

[13] Bleka Øyvind, Prieto L, Gill P (2022) EFMrep: an extension of EuroForMix for improved combination of STR DNA mixture profiles. Forensic Sci International: Genet 61:102771. 10.1016/j.fsigen.2022.102771 10.1016/j.fsigen.2022.102771 36075175

